# Four weeks of regular static stretching reduces arterial stiffness in middle-aged men

**DOI:** 10.1186/s40064-015-1337-4

**Published:** 2015-09-25

**Authors:** Masato Nishiwaki, Haruka Yonemura, Kazumichi Kurobe, Naoyuki Matsumoto

**Affiliations:** Faculty of Engineering, Osaka Institute of Technology, 5-16-1 Ohmiya, Asahi-ku, Osaka, 535-8585 Japan; Faculty of Environmental Symbiotic Sciences, Prefectural University of Kumamoto, Kumamoto, Japan; Faculty of Business, Sports Management Course, Hannan University, Osaka, Japan

**Keywords:** Arteriosclerosis, Brachial-ankle pulse-wave velocity, Cardio-ankle vascular index, Exercise, Flexibility, Sit-and-reach

## Abstract

Trunk flexibility may be associated with arterial stiffness in young, middle-aged, and older healthy men after adjusting for blood pressure. This study assessed the effects of 4 weeks of regular static stretching on arterial stiffness in middle-aged men. Sixteen healthy men (43 ± 3 years) were assigned to control or intervention groups (n = 8 each). The control group did not alter their physical activity levels throughout the study period. The intervention group participated in five supervised stretching sessions per week for 4 weeks. Each session comprised 30 min of mild stretching that moved the major muscle groups through the full range of motion and stretches were held three times for 20 s at the end range. Flexibility was assessed by sit-and-reach test. Arterial stiffness was assessed by brachial-ankle pulse wave velocity (baPWV) and cardio-ankle vascular index (CAVI). Four weeks of stretching increased sit-and-reach (Control, Pre: 31.4 ± 2.1, Post: 30.8 ± 2.7 vs. Intervention, Pre: 30.6 ± 5.3, Post: 43.9 ± 4.3 cm), and reduced baPWV (Control, Pre: 1204 ± 25, Post: 1205 ± 38 vs. Intervention, Pre: 1207 ± 28, Post: 1145 ± 19 cm/s) and CAVI (Control, Pre: 7.6 ± 0.3, Post: 7.5 ± 0.3 vs. Intervention, Pre: 7.7 ± 0.2, Post: 7.2 ± 0.2 units) in the intervention group. However, the change in sit-and-reach did not significantly correlate with the changes in arterial stiffness. These findings suggest that short-term regular stretching induces a significant reduction in arterial stiffness in middle-aged men.

## Background

Pulse wave velocity (PWV) is frequently used as an index of arterial stiffness, and large elastic artery stiffness is progressively greater with advancing age even in healthy people (Avolio et al. 1985). An increase in arterial stiffness is also as an independent risk factor for future cardiovascular disease (Arnett et al. 1994; Laurent and Boutouyrie 2007). Therefore, the prevention and treatment of arterial stiffening are of paramount importance.

Recent studies indicate that a value of sit-and-reach is significantly correlated with brachial-ankle PWV (baPWV), aortic PWV, and cardio-ankle vascular index (CAVI), and poor trunk flexibility is related to greater arterial stiffening (Nishiwaki et al. 2014b; Yamamoto et al. 2009). Furthermore, we have reported that trunk flexibility is related to arterial stiffness independently of blood pressure (BP), which is a major confounding factor (Nishiwaki et al. 2014b). Sex differences may exist in the relationship between trunk flexibility and arterial stiffness (Nishiwaki et al. 2014b). Also, a recent study has indicated that the middle-aged martial artists were more flexible in their trunk and hamstrings and had less arterial stiffness (Douris et al. 2013). Therefore, these findings suggest that some physiological mechanisms, namely structural and/or functional, but not confounding factors, participate in the relationship between trunk flexibility and arterial stiffness.

Regular stretching improves scores in the sit-and-reach test that is an index of trunk flexibility (Kokkonen et al. 2007). If flexibility is physiologically related in some way to arterial stiffness, regular stretching should induce a reduction in arterial stiffness. However, whether stretching per se reduces arterial stiffness without corresponding changes in any other physical characteristics or the physical fitness status of individuals remains a matter of debate (Cortez-Cooper et al. 2008; Hunter et al. 2013a, b; Wong and Figueroa 2014). Because our cross-sectional study indicated a significant relationship between trunk flexibility and arterial stiffness, especially among adult men from young to older (Nishiwaki et al. 2014b), the effects of regular stretching on arterial stiffness in adult men should be evaluated. However, this issue has not been addressed as far as we can ascertain.

This study aimed to examine the effects of 4 weeks of stretching on arterial stiffness in middle-aged men. We hypothesized, based on the findings of our previous cross-sectional study (Nishiwaki et al. 2014b), that regular stretching would reduce arterial stiffness in middle-aged men.

## Results

### Baseline

The mean age, height, body weight, and BMI of all participants were 43 ± 3 years, 172.3 ± 1.3 cm, 69.0 ± 1.6 kg, and 23.3 ± 0.6 kg/m^2^, respectively. Table 1 shows the physical characteristics of the participants in both groups. None of the parameters significantly differed between the control and intervention groups before the study.

**Table 1:** Body composition and cardiovascular data before and after the experimental period

Variables		Pre	Post	Two-way ANOVA		
				Group	Time	Interaction
No.	C	8	-	-	-	-
	I	8	-			
Age, y	C	42 ± 3	-	-	-	-
	I	45 ± 4	-			
Height, cm	C	172.3 ± 2.0	172.0 ± 2.0	F = 0.014	F = 0.636	F = 4.302
	I	172.4 ± 1.8	172.5 ± 1.8	P = 0.908	P = 0.438	P = 0.057
Body weight, kg	C	68.2 ± 2.5	68.5 ± 2.5	F = 0.171	F = 0.083	F = 2.870
	I	69.9 ± 2.2	69.5 ± 2.1	P = 0.685	P = 0.778	P = 0.112
Body mass index, kg/m^2^	C	23.0 ± 0.8	23.1 ± 0.8	F = 0.138	F = 0.115	F = 2.830
	I	23.6 ± 0.9	23.4 ± 0.8	P = 0.716	P = 0.740	P = 0.115
Body fat, %	C	18.7 ± 1.3	18.6 ± 1.2	F = 1.007	F = 0.284	F = 0.339
	I	20.7 ± 2.2	21.3 ± 1.9	P = 0.333	P = 0.602	P = 0.570
Lean body mass, kg	C	55.3 ± 1.3	55.5 ± 1.4	F = 0.087	F = 0.333	F = 1.471
	I	55.2 ± 1.6	54.5 ± 1.1	P = 0.772	P = 0.573	P = 0.245
HR, beats/min	C	61 ± 2	59 ± 3	F = 0.772	F = 2.799	F = 0.465
	I	66 ± 6	62 ± 3	P = 0.394	P = 0.116	P = 0.507
Systolic BP, mmHg	C	129 ± 5	133 ± 3	F = 3.713	F = 2.630	F = 0.006
	I	119 ± 4	123 ± 3	P = 0.075	P = 0.127	P = 0.940
Diastolic BP, mmHg	C	84 ± 4	87 ± 4	F = 2.444	F = 1.362	F = 0.695
	I	78 ± 3	79 ± 2	P = 0.140	P = 0.263	P = 0.419
Mean BP, mmHg	C	99 ± 4	102 ± 3	F = 2.601	F = 0.887	F = 2.016
	I	94 ± 3	93 ± 2	P = 0.129	P = 0.362	P = 0.178
Pulse pressure, mmHg	C	44 ± 3	46 ± 2	F = 0.509	F = 2.215	F = 0.181
	I	41 ± 3	44 ± 3	P = 0.487	P = 0.159	P = 0.677
Handgrip strength, kg	C	45.3 ± 1.8	44.6 ± 1.6	F = 0.217	F < 0.001	F = 0.521
	I	43.6 ± 2.0	44.3 ± 1.1	P = 0.648	P = 0.986	P = 0.482
Handgrip strength, N/kg	C	6.5 ± 0.2	6.4 ± 0.3	F = 1.032	F = 0.059	F = 1.087
	I	6.1 ± 0.2	6.3 ± 0.2	P = 0.327	P = 0.812	P = 0.315

*C* Control group, *I* Intervention group, *HR* Heart rate, *BP* blood pressure, *Pre* before experiment, *Post* after experiment. Data are means ± SEM

No parameters significantly changed in either group after experimental period

### Effects of stretching

**Fig. 1:**
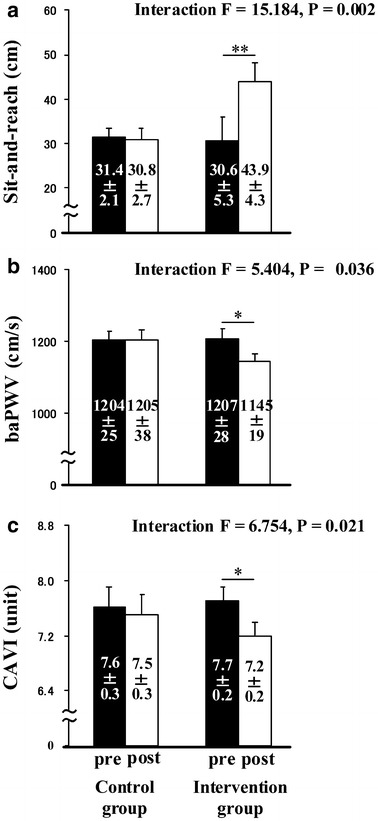
Effects of regular stretching on sit-and-reach (**a**), baPWV (**b**), and CAVI (**c**). *baPWV* brachial-ankle pulse wave velocity that reflects systemic arterial stiffness, *CAVI* cardio-ankle vascular index of arterial stiffness that is theoretically adjusted by blood pressure. Pre, before intervention; Post, after intervention; *p < 0.05 vs. before intervention; **p < 0.01 vs. before intervention. Group × period ANOVA revealed significant interactions among sit-and-reach, baPWV, and CAVI. Short-term regular supervised static stretching induced significant reduction in arterial stiffness

Body composition, BP, HR, and handgrip strength did not significantly change after 4 weeks in either group (Table 1). However, two-way repeated-measures ANOVA indicated significant interactions of sit-and-reach (P < 0.01), baPWV (P < 0.05), and CAVI (P < 0.05) (Fig. 1). Sit-and-reach significantly increased only in the intervention group after 4 weeks of stretching (P < 0.01) (Fig. 1a). Mean baPWV and CAVI values significantly decreased (both P < 0.05) (Fig. 1b, c) in the intervention, but not in the control group. Physical activity levels determined from the IPAQ did not significantly differ between the two groups during the experimental period (control vs. intervention, 26.8 ± 6.3 vs. 15.1 ± 5.8 METs h/week). Both groups answered that physical activity levels had not changed between before and during the intervention period.

### Relationship between trunk flexibility and arterial stiffness

**Fig. 2:**
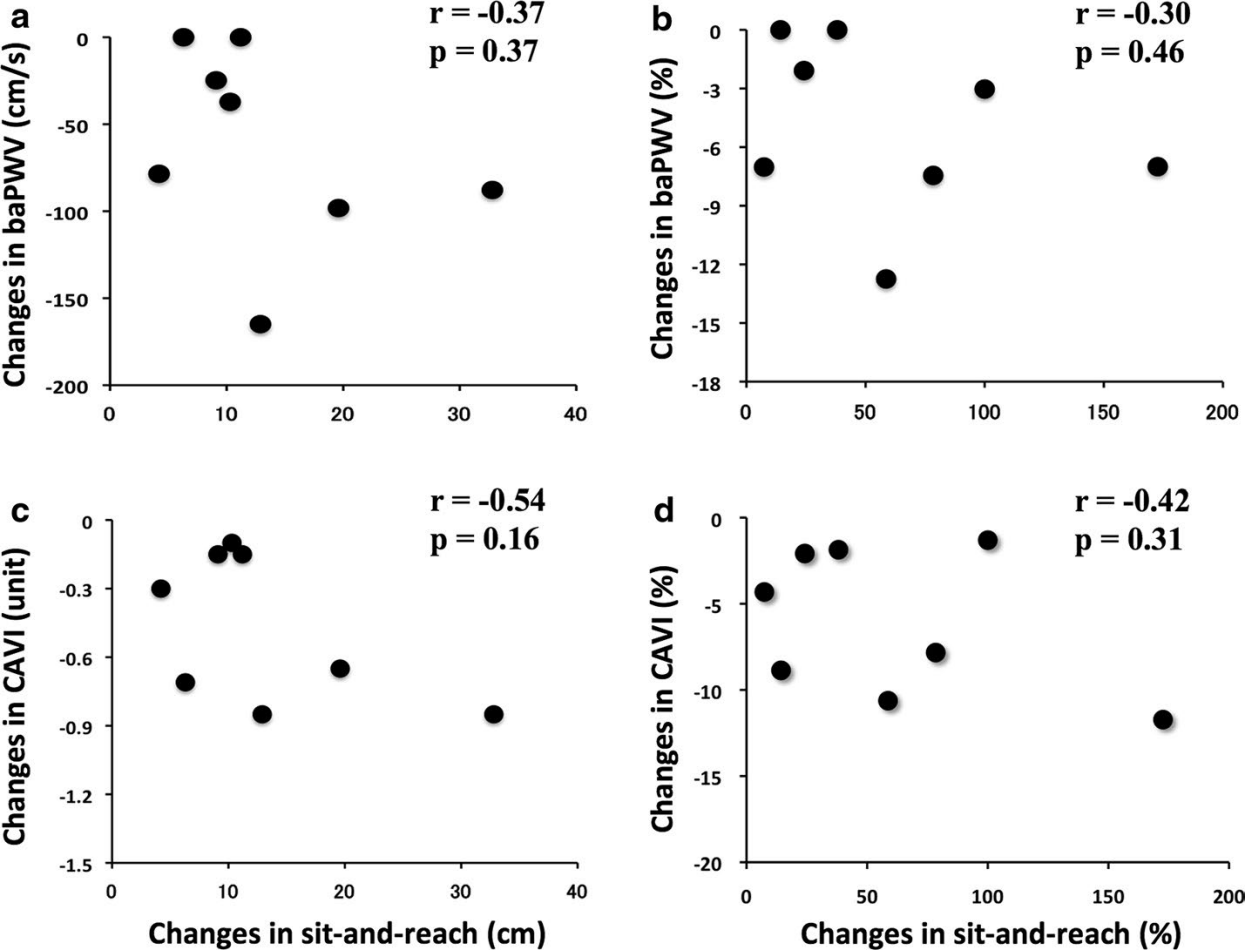
Relationships between changes in sit-and-reach and arterial stiffness. **a** A relationship between changes in sit-and-reach (cm) and baPWV (cm/s) in the intervention group; **b** a relationship between changes in sit-and-reach (%) and baPWV (%) in the intervention group; **c** a relationship between changes in sit-and-reach (cm) and CAVI (unit) in the intervention group; **d** a relationship between changes in sit-and-reach (%) and CAVI (%) in the intervention group. There were no statistically significant correlations between the changes in sit-and-reach and in arterial stiffness

Sit-and-reach increased by 61.7 ± 19.5 %, whereas baPWV and CAVI were reduced by 4.9 ± 1.6 % and 6.1 ± 1.5 %, respectively in the intervention group. The changes in sit-and-reach did not significantly correlate with the changes in arterial stiffness in this group (Fig. 2a–d).

## Discussion

The main new finding of this study was that 4 weeks of supervised static stretching increased sit-and-reach and reduced baPWV and CAVI in middle-aged men without overt chronic diseases. To our knowledge, this is the first study to evaluate the effect of supervised short-term static stretching on arterial stiffness in middle-aged men.

Others have examined the relationship between trunk flexibility and arterial stiffness (Hunter et al. 2013a, b; Nishiwaki et al. 2014b; Wong and Figueroa 2014; Yamamoto et al. 2009), and this relationship may be affected by sex differences (Nishiwaki et al. 2014b). Therefore, the present short-term supervised intervention was designed to determine whether regular stretching exercise can induce a reduction in arterial stiffness in middle-aged men without overt chronic diseases. Our data indicated that short-term stretching reduced baPWV and CAVI, which supported the findings of our previous cross-sectional study (Nishiwaki et al. 2014b). The information derived from baPWV is qualitatively similar to that derived from aortic PWV and carotid-femoral PWV (Sugawara et al. 2005; Tanaka et al. 2009; Vlachopoulos et al. 2012; Yamashina et al. 2002), and also the CAVI represents arterial stiffness from the aorta to the ankle and is theoretically adjusted by BP (Namekata et al. 2011; Shirai et al. 2011; Sun 2013). Because body composition, BP, and handgrip strength did not significantly change before and after intervention in the present study, these results suggest that these factors were not involved in the reduction of baPWV and CAVI that reflect arterial stiffness. Taken together, our results indicate that short-term regular static stretching reduces arterial stiffness.

Although our results mean that flexibility is physiologically related in some way to arterial stiffness, the physiological mechanisms remain unknown. However, previous studies have indicated that muscular-skeletal flexibility is determined by not only the structural factors such as muscle and tendon but also the functional factors such as neural action and reflex, and that regular stretch training may induce neural adjustments (i.e., the decline in tonic reflex activity) (Guissard and Duchateau 2004, 2006; Magnusson et al. 1996). On the other hand, arterial stiffness is functionally determined by the vascular tone of the artery, which is partially regulated by sympathetic nerve activity (Sugawara et al. 2009; Tanaka and Safar 2005; Yamamoto et al. 2009). The neural factors thus contribute to the regulation of body flexibility as well as arterial stiffness. As previously inferred (Yamamoto et al. 2009), repetitive stretching stimulation might chronically reduce resting sympathetic nerve activity, thereby reducing arterial stiffness. A recent study has found that stretching can reduce aortic wave reflection magnitude, BP, and autonomic activity in obese postmenopausal women (Wong and Figueroa 2014), suggesting that regular stretching might affect autonomic activity in normal middle-aged men. Alternatively, stretching might impose traction stimulus to the arteries, resulting in matrix and smooth muscle cell adaptations that favorably affect cross-sectional arterial compliance (Hunter et al. 2013a). However, we did not obtain direct evidence in vivo to support the view. Therefore, future studies are required to elucidate the physiological mechanisms.

Contrary to the present results, 8 weeks of regular stretching does not alter arterial stiffness in obese postmenopausal women (Wong and Figueroa 2014). Cross-sectional and interventional studies have also found that the regular practice of hatha yoga does not change arterial compliance (Hunter et al. 2013b). The cause of these conflicting findings between the present and previous studies is presently difficult to explain. However, our cross-sectional study indicated that poor trunk flexibility may be significantly related to arterial stiffening, especially in adult men (Nishiwaki et al. 2014b). In addition, yoga, especially in hatha yoga postures, includes a component of isometric contractions (Hunter et al. 2013a, b). Although isometric exercise can reduce BP and increase endothelial function (McGowan et al. 2007), the combined effects of stretching and isometric contractions may result in the conflicting findings between the present and previous studies. Therefore, stretching-induced vascular adaptations would differ according to the features of the participants such as age, sex, and medical history, as well as the mode, intensity, duration, frequency, and volume of exercise programs.

Regular aerobic exercise reduces arterial stiffness in middle-aged people (Guimaraes et al. 2010; Yoshizawa et al. 2009), and previous study indicates that 16 weeks of aerobic exercise induces approximately 5 % reduction in carotid-femoral PWV (Guimaraes et al. 2010). On the other hand, low-intensity resistance training for young subjects induces approximately 6.7 % reduction in baPWV (Okamoto et al. 2011). Home-based resistance training using body weight and light dumbbells also improves arterial stiffness in health middle-aged women (Okamoto et al. 2009). The present data showed that 4 weeks of supervised stretching exercise produced a 4.9 ± 1.6 % reduction in baPWV and a 6.1 ± 1.5 % reduction in CAVI as an index of arterial stiffness. The duration of stretching was relatively short; nevertheless, our stretching intervention may generally induce similar or somewhat smaller changes in arterial stiffness compared with relatively low- to moderate-intensity aerobic or resistance exercise for middle-aged people.

Although our stretch training intervention consisted of whole body stretching, the present study only used the sit-and-reach test as an indicator of flexibility. This test might be differentially influenced by arm and leg length, but the individual zero point for each participant minimized the influences of arm and leg length. Thus, the sit-and-reach test has been commonly used to assess flexibility from the viewpoint of health-related fitness (Yamamoto et al. 2009). Although our data of sit-and-reach test are actually considered to reflect trunk flexibility, further studies will be needed to assess the flexibility in trunk and other muscle groups during postural control using electromyography and goniometer.

This study has several important limitations. First, although we calculated the minimum sample size, the number of participants might have been too small to detect low-to-moderate correlations between changes in flexibility and arterial stiffness. Further large-scale studies are required to clear this point. Second, we could not assess maximum oxygen uptake and blood data and thus the cardiorespiratory fitness levels of the participants and risk factors for atherosclerosis such as blood lipids were unknown. Furthermore, in this study, the body composition did not change significantly after the intervention, and the IPAQ score did not differ significantly between the both groups. However, if possible, physical activity should be assessed by objective measures such as activity monitor or pedometer.

## Conclusions

The present findings indicated that 4 weeks of regular static stretching induces a significant reduction in arterial stiffness in middle-aged men. Although this reduction in arterial stiffness did not significantly correlate with the increase in trunk flexibility, in order to clarify the relationship between the change in flexibility and arterial stiffness in more detail, further large-scale studies are needed.

## Methods

### Participants

Sixteen middle-aged Japanese men without chronic diseases that could affect cardiovascular, metabolism, or daily physical activity were recruited for the present study from among the staff at our institution. They were regularly engaged in desk work inside of the office (>8 h/day), and none of them participated in regular exercise program for at least 2 years. The participants did not take any medications. The men were matched for age and physical characteristics, especially in PWV as nearly equal as possible and assigned to either a control or an intervention group (n = 8 each). Thus, the participants were not provided with the option to self-select their group allocation. The purpose, procedures, and risks of the study were explained to all of them and they provided written informed consent before participating in the study, which was reviewed and approved by the Human Ethics Committee at the Prefectural University of Kumamoto (24-002) and proceeded in accordance with the guidelines of the Declaration of Helsinki.

### Sample size and experimental procedures

We determined the appropriate sample size for each group before starting the study by power calculations using SPSS Sample Power (IBM, Tokyo, Japan) and assumed that the maximal reduction in arterial stiffness after intervention would be 4–7 % according to the previous results (Nishiwaki et al. 2014b; Yamamoto et al. 2009). To detect this difference at 80 % power and with a two-tailed α of 5 %, the intervention group should comprise eight participants. Thus, we recruited 16 participants and assigned them to control and intervention groups (n = 8 per group).

Both groups were assessed before and after the experimental period. All tests and interventions proceeded in a quiet air-conditioned room (22–24 °C) at the same time of day and at the same number of hours after the last meal to avoid potential diurnal variations. The participants were required to abstain from caffeine and fast for ≥4 h before each test and then were assessed at least 24 h after the most recent stretching session to avoid any acute effects.

### Body composition

Body composition was determined by bioelectrical impedance using a TBF-410 instrument (Tanita Co., Tokyo, Japan) as described in our previous study (Nishiwaki et al. 2014a, b). Briefly, we measured the height of the participants without footwear, weight, and body fat in light clothing, and then calculated their body mass index (BMI), as weight divided by height squared. This method can accurately detect changes in body composition and the validity of body composition assessment is high (Miyatani et al. 2012; Utter et al. 1999). Day-to-day coefficients of variations (CVs) for body weight, body fat, and BMI were all <10 % under our experimental conditions (Nishiwaki et al. 2014a, b).

### Arterial stiffness, blood pressure, and heart rate

After resting for ≥15 min in the supine position, PWV, BP and heart rate (HR) were assessed using an automated VS-1500AE/AN device (Fukuda Denshi, Tokyo, Japan) as described (Namekata et al. 2011; Shirai et al. 2011; Sun 2013). This device records BP in both brachial locations of supine participants and the procedure conformed strictly to American Heart Association guidelines (Pickering et al. 2005). As previously reported (Namekata et al. 2011; Shirai et al. 2011; Sun 2013), the CAVI values at the right and left sides were also automatically calculated from 5 to 6 pulse wave signals using the following formula: ${\text{CAVI}} \; = \; {\text{a }}[\left( { 2\rho /{\text{PP}}} \right)\; \times \; { \ln }\left( {{\text{SBP}}/{\text{DBP}}} \right) \; \times \; {\text{PWV2}}] \; + \; {\text{b}}$, where SBP is systolic blood pressure, DBP is diastolic blood pressure, PP (pulse pressure) is SBP − DBP, ρ is the blood density, and a and b are constants. The means of the left and right values in each participant were subsequently analyzed (Nishiwaki et al. 2014b; Yamamoto et al. 2009). The CAVI represents arterial stiffness from the aorta to the ankle and is theoretically adjusted by BP. Higher CAVI values mean stiffer arteries and the CAVI is, therefore, associated with risk for cardiovascular diseases with excellent validity (Shirai et al. 2011; Sun 2013). The baPWV was also calculated by dividing the distance between the brachial and ankle recording sites by the transit times as described (Nishiwaki et al. 2011; Yamashina et al. 2002). Previous studies have indicated that the information derived from baPWV is qualitatively similar to that derived from central arterial stiffness and that the validity and reproducibility of baPWV measurements are high (Sugawara et al. 2005; Tanaka et al. 2009; Yamashina et al. 2002). The CVs from the intraobserver measured over 10 subjects on two separate days (i.e., reproducibility) were 3.6 ± 0.6 % and 2.7 ± 0.3 % for CAVI and baPWV, respectively (Nishiwaki et al. 2011, 2014b).

### Trunk flexibility

Trunk flexibility was assessed by measuring a sit-and-reach test using a T-283 device (Toei Light, Tokyo, Japan) as described at least twice after stretching (Nishiwaki et al. 2014b; Yamamoto et al. 2009), and then the average of the two highest values was taken as the definitive value (CV, 6.4 ± 1.6 %).

### Handgrip strength

Handgrip strength was measured using a T.K.K.5001 Grip-A dynamometer (Takei, Tokyo, Japan) with a precision of 0.1 kg (CV, 4.1 ± 0.8 %). Measurements were made in duplicate, and the highest value in the stronger hand was taken (Cappola et al. 2001; Leenders et al. 2013). In addition, handgrip strength data were normalized to body weight (Nishiwaki et al. 2014b).

### Physical activity levels

Habitual physical activity levels during the experimental period were assessed using the validated, reliable International Physical Activity Questionnaire (IPAQ short form) translated into Japanese (Craig et al. 2003; Miura et al. 2012; Nishiwaki et al. 2014b). The intervention group answered questions about regular physical activity other than stretching intervention. We also interviewed the participants to determine whether physical activity levels had not changed during the experimental period compared with before (yes, 1; no, 2).

### Stretch training

Supervised mild stretching was performed for approximately 30 min per training session, five different days per week, for 4 weeks in a room with temperature controlled at 22–24 °C according to a study showing that hamstring flexibility can increase during this time frame (Cipriani et al. 2012). Each training session was performed between 17:30 and 19:30. Expert instructors demonstrated the stretches for the participants throughout the experimental period. The instructors designed the stretches to take the major muscle groups, namely the lower extremities (quadriceps, hamstring, adductor, and gastrocnemius muscles), upper extremities (pectoralis major and minor, triceps, latissimus dorsi), and the neck and trunk (flexion, extension, and rotation) through their full range of motion as described (Cortez-Cooper et al. 2008). The program consisted of one site of 3 bouts of 20 s stretching at the end range (point of minimal discomfort) with approximately 30–40 s rest between each bout. All participants in the intervention group completed 100 % of all scheduled stretching sessions (20 sessions over a period of 4 weeks). The participants in the both groups were instructed to maintain their normal diet and to refrain from any other specific exercise training throughout the study period.

### Statistics

Results are presented as mean ± standard error of the mean (SEM). Bartlett’s test and Levene’s test were firstly performed to confirm the normal distribution of the data. Parameters before the experiment and physical activity levels between the two groups were compared using an independent Student’s *t* test. Changes in parameters were analyzed by a two-way (group × period) repeated-measures analysis of variance (ANOVA). When the F value was significant, the Bonferroni method was applied for post hoc multiple comparisons. Relationships between flexibility and arterial stiffness were assessed using Pearson’s correlation. Results were regarded as statistically significant at P < 0.05.

## Abbreviations

ANOVA: analysis of variance
BMI: body mass index
BP: blood pressure
baPWV: brachial-ankle PWV
CAVI: cardio-ankle vascular index
CV: coefficients of variations
DBP: diastolic blood pressure
HR: heart rate
IPAC: International physical activity questionnaire
PP: pulse pressure
PWV: pulse wave veloxity
SBP: systolic blood pressure
SEM: standard error of the mean
